# miR-4775 promotes colorectal cancer invasion and metastasis via the Smad7/TGFβ-mediated epithelial to mesenchymal transition

**DOI:** 10.1186/s12943-017-0585-z

**Published:** 2017-01-17

**Authors:** Senlin Zhao, Hongcheng Sun, Weiliang Jiang, Yushuai Mi, Dongyuan Zhang, Yugang Wen, Dantong Cheng, Huamei Tang, Shaohan Wu, Yang Yu, Xisheng Liu, Weiyingqi Cui, Meng Zhang, Xiaofeng Sun, Zongguang Zhou, Zhihai Peng, Dongwang Yan

**Affiliations:** 1 Department of General Surgery, Shanghai General Hospital, School of Medicine, Shanghai Jiao Tong University, 85 Wujin Road, Shanghai, 200080 China; 2 Department of Gastroenterology, Shanghai General Hospital, School of Medicine, Shanghai Jiao Tong University, 85 Wujin Road, Shanghai, 200080 China; 3 Department of Pathology, Shanghai General Hospital, School of Medicine, Shanghai Jiao Tong University, 85 Wujin Road, Shanghai, 200080 China; 4 Department of General Surgery, The Second Affiliated Hospital of Jiaxing College, 1518 Huancheng North Road, Jiaxing, Zhejiang 314000 China; 5 Department of Oncology and Department of Clinical and Experimental Medicine, Linköping University, SE-581 83 Linköping, Sweden; 6 Department of Pathology, Fudan University, Shanghai Cancer Center, 270 Dongan Road, 200030 Shanghai, China; 7 Department of Gastrointestinal Surgery, West China Hospital, Sichuan University, No. 37 Guo Xue Xiang Street, Chengdu, 610041 Sichuan China

**Keywords:** miR-4775, Smad7, TGFβ signaling, Colorectal cancer, EMT

## Abstract

**Background:**

Despite advancements in the diagnosis and treatment of colorectal cancer (CRC), many patients die because of tumor metastasis or recurrence. Therefore, identifying new prognostic markers and elucidating the mechanisms of CRC metastasis and recurrence will help to improve the prognosis of the disease. As dysregulation of microRNAs is strongly related to cancer progression, the aim of this study was to identify the role of miR-4775 in the prognosis of CRC patients and the underling mechanisms involved in CRC progression.

**Methods:**

qPCR and in situ hybridization were used to evaluate the expression of miR-4775 in 544 pairs of paraffin-embedded normal and CRC tissues. Kaplan–Meier analysis with the log-rank test was used for survival analyses. Immunohistochemical staining was applied to investigate the expression of miR-4775-regulated Smad7/TGFβ pathway-associated markers. In vitro and in vivo invasion and metastasis assays were used to explore the function of miR-4775 in the progression of CRC.

**Results:**

miR-4775 was identified as a high-risk factor for CRC metastasis and recurrence, with high levels predicting poor survival among the 544 studied CRC patients. Furthermore, high miR-4775 expression promoted the invasion of CRC cells as well as metastasis and the epithelial to mesenchymal transition (EMT) via Smad7-mediated activation of TGFβ signaling both in vitro and in vivo. Downregulating miR-4775 or overexpressing Smad7 reversed the tumor-promoting roles of miR-4775/Smad7/TGFβ in vitro and in vivo.

**Conclusion:**

miR-4775 promotes CRC metastasis and recurrence in a Smad7/TGFβ signaling-dependent manner, providing a new therapeutic target for inhibiting the metastasis or recurrence of the disease.

**Electronic supplementary material:**

The online version of this article (doi:10.1186/s12943-017-0585-z) contains supplementary material, which is available to authorized users.

## Background

Colorectal cancer (CRC) is the fourth leading cause of cancer-related mortality worldwide [1], and tumor invasion and metastasis are the main causes of mortality in these patients [2]. During invasion and metastasis, activation of oncogenes and inactivation of suppressors play significant roles [3]. Although many mechanisms underlying these processes have been elucidated and treatment has improved, 50% of CRC patients still develop metastases following surgery [4]. Therefore, identification of new predictive markers and the mechanisms involved in the invasion and metastasis of CRC is urgently needed.

MicroRNAs (miRNAs) are small non-coding RNAs (18–24 nucleotides) that can exist stably in serum and mediate biological processes such as tumor cell migration, invasion, proliferation and apoptosis [5]. Increasing evidence has demonstrated that aberrant expression of miRNAs can directly promote or inhibit CRC progression by degrading the 3′ untranslated regions (3′UTRs) of target genes [6]. For example, miR-34a-5p suppresses CRC metastasis via a p53-dependent pathway, and low miR-34a-5p expression was found to predict good prognosis in stage II/III CRC patients [3]. Through transforming growth factor (TGFβ), miR-1269a directly targets Smad7 and HOXD10, forming a positive feedback loop that enhances the metastatic capacity of CRC cells [7]. In addition, miR-4775 has been verified as a new onco-miRNA with a dual effect on the ERBB2/Her2 gene [8]. However, there are no reports to date on the function of miR-4755 in CRC progression.

The Smad7 gene, also known as mothers against decapentaplegic homolog 7 (MADH7), is located on human chromosome 18q21.1 and encodes a protein of 426 amino acids [9]. Smad7-dependent activation of TGFβ signaling is able to induce the epithelial to mesenchymal transition (EMT) in CRC, allowing CRC cells to leave the tissue parenchyma and enter systemic circulation, followed by tumor invasion and metastasis [10]. Smad7 is an inhibitory Smad protein that interacts with TGFβ type I receptor, targeting it for proteasomal degradation and thereby inhibiting TGFβ-I-induced phosphorylation of Smad2/Smad3 [11]. Smad7 usually acts as a tumor suppressor [12]. Indeed, it has been reported that Smad7 inhibits breast cancer metastasis to the lung and liver, represses melanoma cell metastasis to bone, and inhibits hepatocellular carcinoma cell proliferation and invasion [13–15]. According to gene target prediction, a miR-4775 recognition sequence is present in the Smad7 3′UTR, though it remains unclear whether miR-4775 promotes CRC metastasis through activation of the Smad7/TGFβ pathway.

In this study, we verified the following: 1) miR-4775 is more highly expressed in metastatic CRC tissues than in non-metastatic tumor tissues; 2) high miR-4775 expression predicts poor prognosis in CRC patients; 3) miR-4775 promotes CRC cell invasion, metastasis and EMT by activating Smad7-dependent TGFβ signaling; and 4) miR-4775-mediated Smad7/TGFβ activation predicts poor survival in CRC patients. These data clarify the role of miR-4775 in CRC progression and provide a new marker for predicting CRC metastasis.

## Methods

### Patient tissue samples and tissue microarray construction

A total of 544 patients with CRC diagnosed at the Department of General Surgery, Shanghai General Hospital, School of Medicine, Shanghai Jiaotong University from 2005 to 2011 were enrolled. None of the patients received anti-cancer treatment prior to tumor resection, and those with advanced-stage disease received standard postoperative 5-fluorouracil-based chemotherapy. Based on their tumor recurrence status, we divided the 544 patients into tumor recurrence and non-recurrence groups (Additional file 1: Table S1). Tissue samples were obtained during operations. This research was approved by the Ethics Committee of our hospital, and informed consent was obtained from all patients before being enrolled in the study. As previously reported, we constructed a tissue microarray that included samples from these 544 patients [16].

### Cell culture and transfection

We purchased CRC (SW480, HT-29, DLD-1, HCT116, RKO, LoVo, HCT8, and SW62) and normal colon epithelial (FHC) cell lines from the American Type Culture Collection. All cell lines were tested by short tandem repeat analysis and used within 6 months; the last time of authentication was February 2016. All cells were cultured in Dulbecco’s modified Eagle’s medium (Gibco BRL, Grand Island, NY, USA) containing 10% fetal bovine serum (FBS, Invitrogen, Camarillo, CA, USA). Cells in which miR-4775 was either overexpressed or knocked down were generated by transfecting miR-4775 mimics and anti-miR-4775 mimics, respectively. Smad7 was overexpressed or knocked down by transfecting with lv-Smad7 or shRNA Smad7 plasmids, respectively. The empty vector and negative control (NC) vector were used as controls for miR-4775 mimics and anti-miR-4775 mimics, respectively. NC and a scrambled vector were used as controls for the lv-Smad7 and sh-Smad7 vector, respectively. All overexpression and knockdown sequences used in the study were the same as those in a previous report [17].

### qRT-PCR, in situ hybridization and western blotting

We performed a quantitative reverse transcription polymerase chain reaction (qRT-PCR) assay, in situ hybridization, and western blotting as described previously [13, 14, 18, 19]. U6 and GAPDH were used as internal controls. Antibody data are summarized in Additional file 2: Table S2.

### In vitro wound healing, migration and invasion assays

For the wound healing assay, we plated cells into six-well plates and scraped the cells when they grew to cover 90% of the surface. The cells were washed after 48 h, and the closed wound widths were then measured and analyzed.

Matrigel invasion chambers (8.0 μm pore size, BD Biosciences, Franklin Lakes, NJ, USA) and Transwell cell migration plates were used for migration and invasion assays. In total, 5 × 10^5^ cells/ml were seeded in serum-free medium in the upper chamber; 10% FBS was added to the lower chamber. For the invasion assay, the Matrigel coating was overlaid in the upper chamber. The cells were incubated for 24 h and fixed, and 0.1% crystal violet was used to stain the cells that adhered to the underside of the membrane, which were counted under a light microscope (Olympus Corporation, Center Valley, PA).

### Immunofluorescence assay

Cells were incubated overnight in glass-bottom dishes, washed three times with phosphate-buffered saline (PBS), fixed with 4% paraformaldehyde for 15 min and washed three times with PBS. The cells were then permeabilized with 0.1% Triton X-100 for 10 min and incubated overnight at 4 °C with primary antibodies against E-cadherin, N-cadherin, and vimentin. After washing, Alexa Fluor 488-conjugated secondary antibodies (Santa Cruz Biotechnology, Santa Cruz, CA) were added for 2 h at room temperature. Nuclei were stained for 5 min with 4′,6-diamidino-2-phenylindole (DAPI; Roche, Basel, Switzerland). A confocal laser-scanning microscope (TCS SP8; Leica, Wetzlar, Germany) was used to collect fluorescence images.

### In vivo tumorigenesis in nude mice

To explore metastasis ability in vivo, cells overexpressing or knocked down for miR-4775 and control cancer cells (10^5^/ml, 200 μl) were injected into the tail veins of nude mice. All mice were sacrificed after 4 weeks. The tumor colonies formed in the lungs and livers were observed and counted under a microscope using hematoxylin-eosin (HE) staining.

### Statistical analysis

For categorical variables, either Fisher’s exact test or a Chi-square test was used to compare differences. For continuous variables, either Student’s t-test or one-way analysis of variance was used to analyze differences. Kaplan–Meier analyses with log-rank tests were used to evaluate disease-free survival (DFS) and overall survival (OS). The Cox proportional hazard model was applied to estimate the hazard ratio and 95% confidence intervals for DFS and OS. SPSS 22.0 statistical software (SPSS Inc., Chicago, IL) was used for the data analysis. A *p* value < 0.05 indicates a significant difference.

## Results

### Elevated miR-4775 is correlated with CRC progression and indicates poor survival for CRC patients

**Fig. 1:**
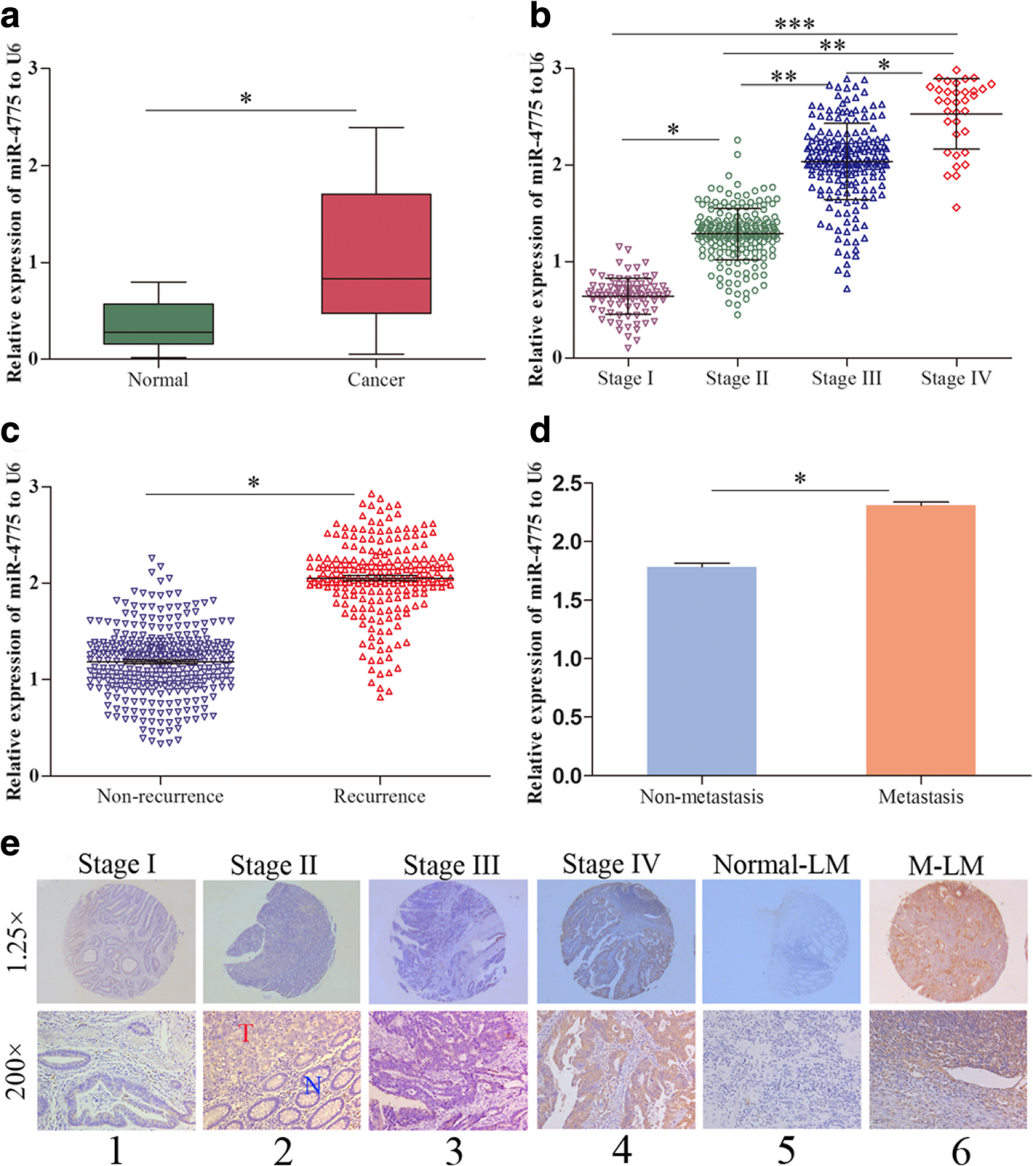
High expression of miR-4775 is correlated with tumor progression in CRC patients. **a** qRT-PCR analyses of miR-4775 expression in 544 pairs of normal and CRC paraffin-embedded tissues (*p < 0.05). **b** Relative miR-4775 expression in stage I to stage IV tumor tissues (*p < 0.05, **p < 0.01, ***p < 0.001). **c** Relative miR-4775 expression in tumor recurrence and non-recurrence groups (*p < 0.05). **d** Relative miR-4775 expression in metastasis and non-metastasis patients (*p < 0.05). **e** miR-4775 in situ hybridization (ISH) in a tissue microarray including 544 pairs of normal and tumor tissues. T, tumor tissue; N, normal tissue; normal-LM, lymph node without tumor metastasis; M-LM, lymph node with tumor metastasis

To investigate the role of miR-4775 in CRC progression, we performed qRT-PCR and in situ hybridization (ISH) on 544 pairs of paraffin-embedded normal and CRC tissues. We found that miR-4775 was significantly upregulated in CRC tissues compared to normal tissues (Fig. 1a and e2). We then compared the expression level of miR-4775 in CRC tissues at different stages and found that levels gradually increased from stage I to stage IV (Fig. 1b and e1–4). These analyses also showed that miR-4775 was more highly expressed in the tumor recurrence group than in the non-recurrence group (Fig. 1c). More importantly, higher miR-4775 expression was observed in patients with metastasis compared to those without metastasis (Fig. 1d and e5–6). Taken together, these results indicate that miR-4775 has important functions in CRC tumorigenesis and metastasis.

Next, we examined the correlation between miR-4775 expression and TNM stage in 544 CRC patients. In total, 402 (73.90%) of CRC patient tissues showed high miR-4775 expression (Additional file 3: Table S3), and high miR-4775 expression was positively associated with T-stage, lymph node metastasis, and distant metastasis (Additional file 3: Table S3). These results further demonstrate that upregulated miR-4775 promotes CRC progression and might play a significant role in the prognosis of CRC patients.

**Fig. 2:**
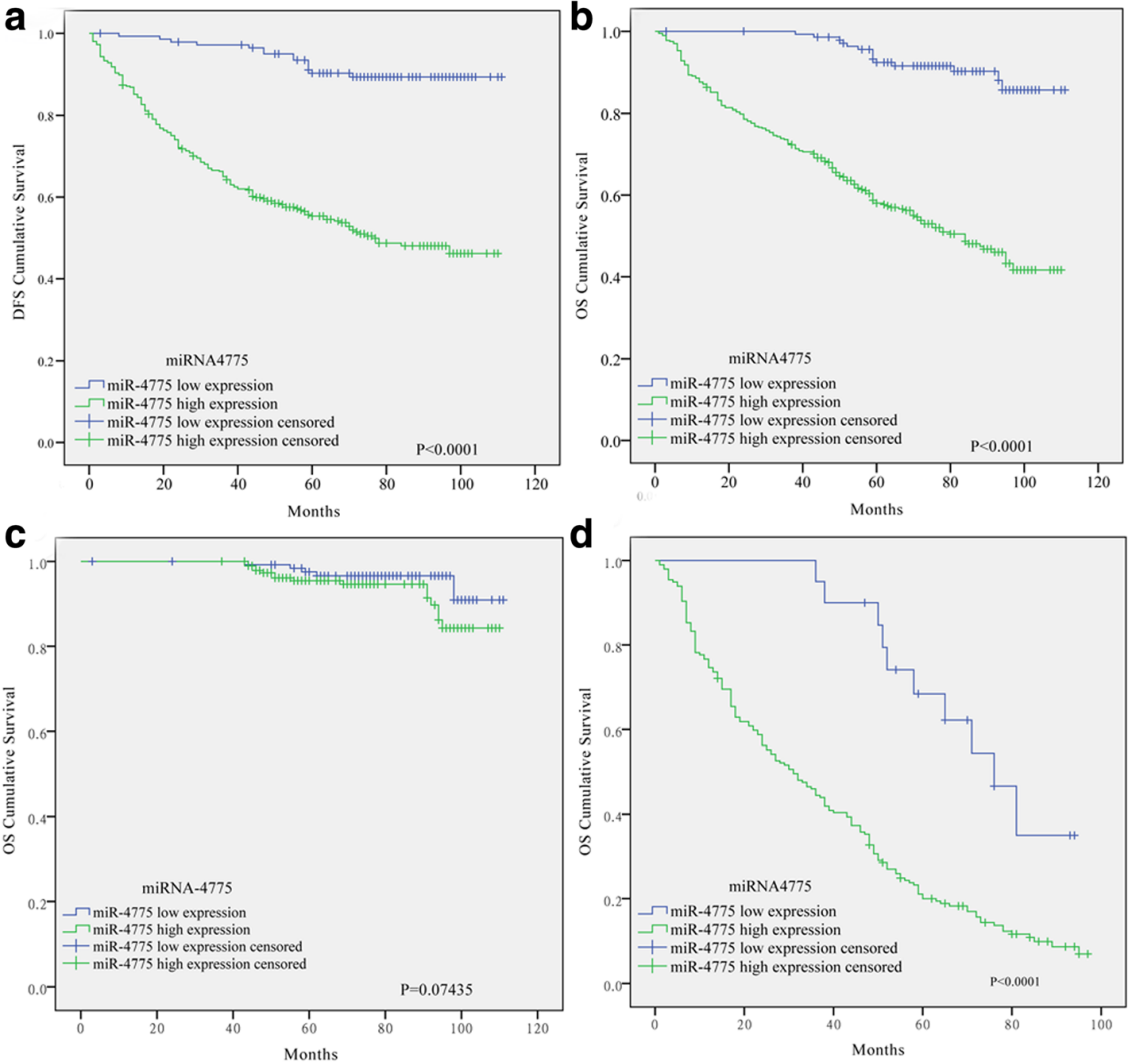
High miR-4775 expression predicts poor survival in CRC patients, especially for tumor recurrence patients. **a** Kaplan–Meier analysis with a log-rank test for DFS in 544 CRC patients according to miR-4775 expression. **b**–**d** Kaplan–Meier analysis with a log-rank test for OS in all 544 patients (**b**) as well as non-recurrence (**c**) and recurrence groups (**d**). High miR-4775 predicts worse OS, especially in tumor recurrence patients

Next, Kaplan–Meier analysis with the log-rank test was used to evaluate the ability of miR-4775 to predict survival in the 544 CRC patients. Patients with a high level of miR-4775 showed lower DFS and OS rates than those with a low level of miR-4775 (Fig. 2a and b). Interestingly, no significant difference between high and low miR-4775 expression was observed for OS in the non-recurrence group (Fig. 2c), whereas high miR-4775 expression did predict poorer OS in the recurrence group (Fig. 2d). Furthermore, multivariate Cox proportional hazard analysis suggested that miR-4775 is an independent prognostic marker for tumor progression and metastasis in CRC patients (Additional file 4: Table S4).

### miR-4775 is involved in EMT and Smad7/TGFβ pathway activation in CRC patients

**Fig. 3:**
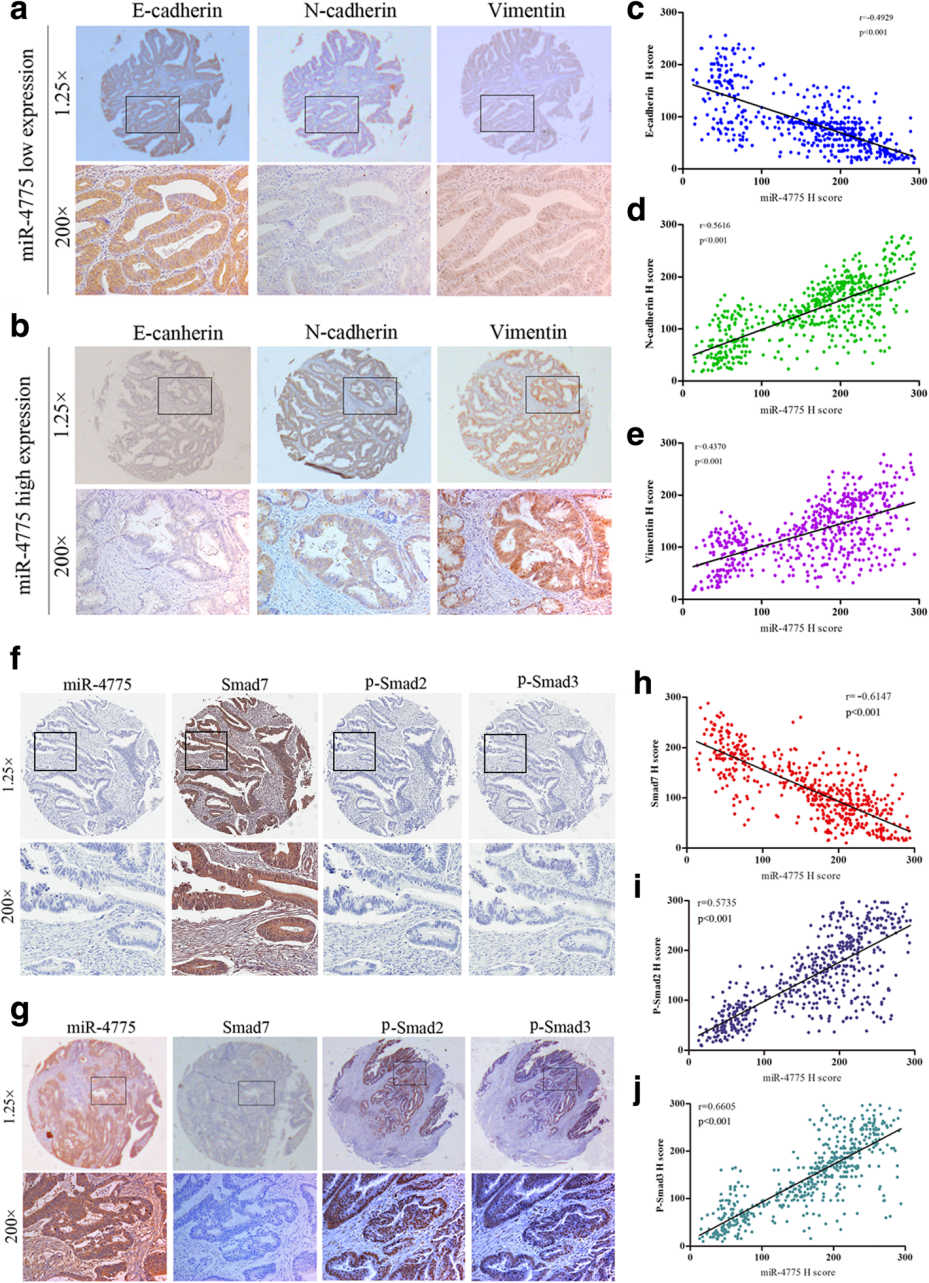
High miR-4775 expression is positively correlated with EMT and Smad7/TGFβ pathway activation in CRC patients. **a**–**b** Representative IHC staining analyses of E-cadherin, N-cadherin, and vimentin in CRC tumor tissues with low or high miR-4775 expression (**c**–**e**). Spearman’s correlation analyses showed that miR-4775 was negatively associated with E-cadherin expression but positively correlated with N-cadherin and vimentin expression in 544 CRC tumor tissues (*p* < 0.001 for all). **f**–**g** Representative IHC staining of Smad7, p-Smad2 and p-Smad3 in pathologic serial sections of CRC tumor tissues with low and high miR-4775 expression. **h**–**j** Spearman’s correlation analyses among miR-4775, Smad7, p-Smad2 and p-Smad3 H-scores in 544 CRC tumor tissues

EMT is an important process during cancer progression, conferring epithelial cancer cells with increased invasiveness and motility [20]. During the EMT process, the cancer cell phenotype typically changes from epithelial to mesenchymal, followed by upregulation of an epithelial marker (E-cadherin) and downregulation of mesenchymal markers (e.g., N-cadherin, vimentin) [21]. Accordingly, we employed immunohistochemical staining (IHC) to analyze the expression of E-cadherin, N-cadherin, and vimentin in tumor tissue from 544 CRC patients. The results showed frequent co-incidence of low E-cadherin expression and high N-cadherin and vimentin expression in tumor tissues highly expressing miR-4775. Conversely, high expression of E-cadherin and low expression of N-cadherin and vimentin were generally found in tumor tissues of patients with low levels of miR-4775 expression (Additional file 5: Table S5, Fig. 3a and b). Pearson’s correlation analyses based on IHC staining scores revealed a negative correlation between miR-4775 and E-cadherin expression (*r* = −0.4929, *p* < 0.001, Fig. 3c) but a positive association between miR-4775 and N-cadherin (*r* = 0.5616, *P* < 0.001, Fig. 3d) and vimentin (*r* = 0.4370, *p* < 0.001, Fig. 3e) expression. These results show that miR-4775 expression is positively associated with EMT in CRC.

The Smad7/TGFβ pathway is involved in EMT and CRC progression [17, 22, 23]. Thus, we further investigated whether miR-4775 is positively correlated with activation of Smad7/TGFβ signaling in CRC tissues. IHC staining of the same patients’ tissues showed that high miR-4775 expression was usually concomitant with weak Smad7 staining but strong p-Smad2 and p-Smad3 staining (Fig. 3f, Additional file 6: Table S6); in contrast, low miR-4775 expression was often concomitant with strong Smad7 staining but weak p-Smad2 and p-Smad3 staining (Fig. 3g, Additional file 6: Table S6). Furthermore, Pearson’s correlation analysis revealed a negative correlation between miR-4775 expression and Smad7 staining but a positive correlation with p-Smad2 and p-Smad3 staining in CRC tissues (*p* < 0.05 for all, Fig. 3h–g). Taken together, these results show that miR-4775 is not only involved in EMT but is also correlated with Smad7/TGFβ pathway activation in the progression of CRC.

### miR-4775 promotes CRC cell invasion and metastasis in vitro and in vivo

**Fig. 4:**
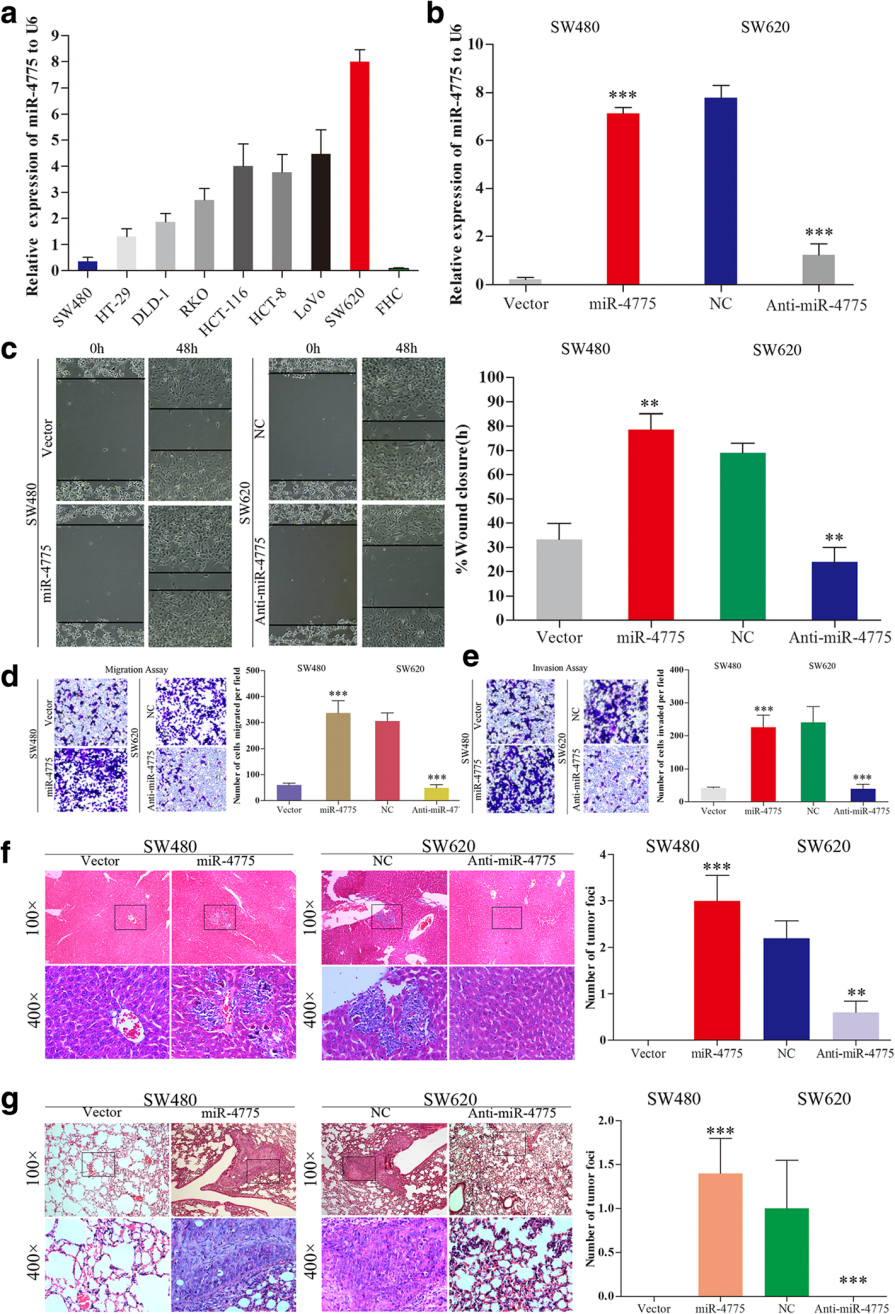
miR-4775 promotes CRC cell invasion and metastasis in vitro and in vivo. **a** Relative miR-4775 expression in 8 CRC cell lines and one normal colon epithelial line (FHC). **b** miR-4775 was overexpressed or knocked down by transfecting miR-4775 mimics or anti-miR-4775 vectors (***p < 0.001). **c**–**e** Overexpression or knockdown of miR-4775 increased or decreased, respectively, CRC cell wound healing, migration and invasion abilities in vitro (**p < 0.01,***p < 0.001). **f**–**g** CRC cells in lung and liver metastases in nude mice. Upregulation and downregulation of miR-4775 increased and decreased, respectively, CRC lung and liver metastases in vivo (**p < 0.01, ***p < 0.001)

To explore the underlying biological mechanisms of miR-4775 function in CRC progression, we evaluated miR-4775 expression in 8 CRC cell lines and one normal epithelial cell line (FHC). Among the 8 CRC cell lines, SW620 and SW480 cells showed the highest and lowest miR-4775 expression, respectively, compared with FHC cells (Fig. 4a). Thus, we chose the SW480 and SW620 cell lines for further investigation and generated cells overexpressing and knocked down for miR-4775, respectively (Fig. 4b). A wound healing assay showed that miR-4775 overexpression or knockdown promoted or delayed wound closure, respectively, compared with the respective control (*p* < 0.01 for both, Fig. 4c). In addition, in vitro migration and invasion assays demonstrated that miR-4775 overexpression or knockdown resulted in higher or lower rates, respectively, of CRC cell migration and invasion compared with the controls (*p* < 0.001 for all, Fig. 4d and e). Consistent with these in vitro results, overexpressing or knocking down miR-4775 expression increased or decreased, respectively, lung and liver metastasis in nude mice compared with the control groups (*p* < 0.001 for all, Fig. 4f and g). Taken together, the results show that miR-4775 increases the invasion and metastatic ability of CRC cells both in vitro and in vivo.

### miR-4775 induces EMT in CRC

**Fig. 5:**
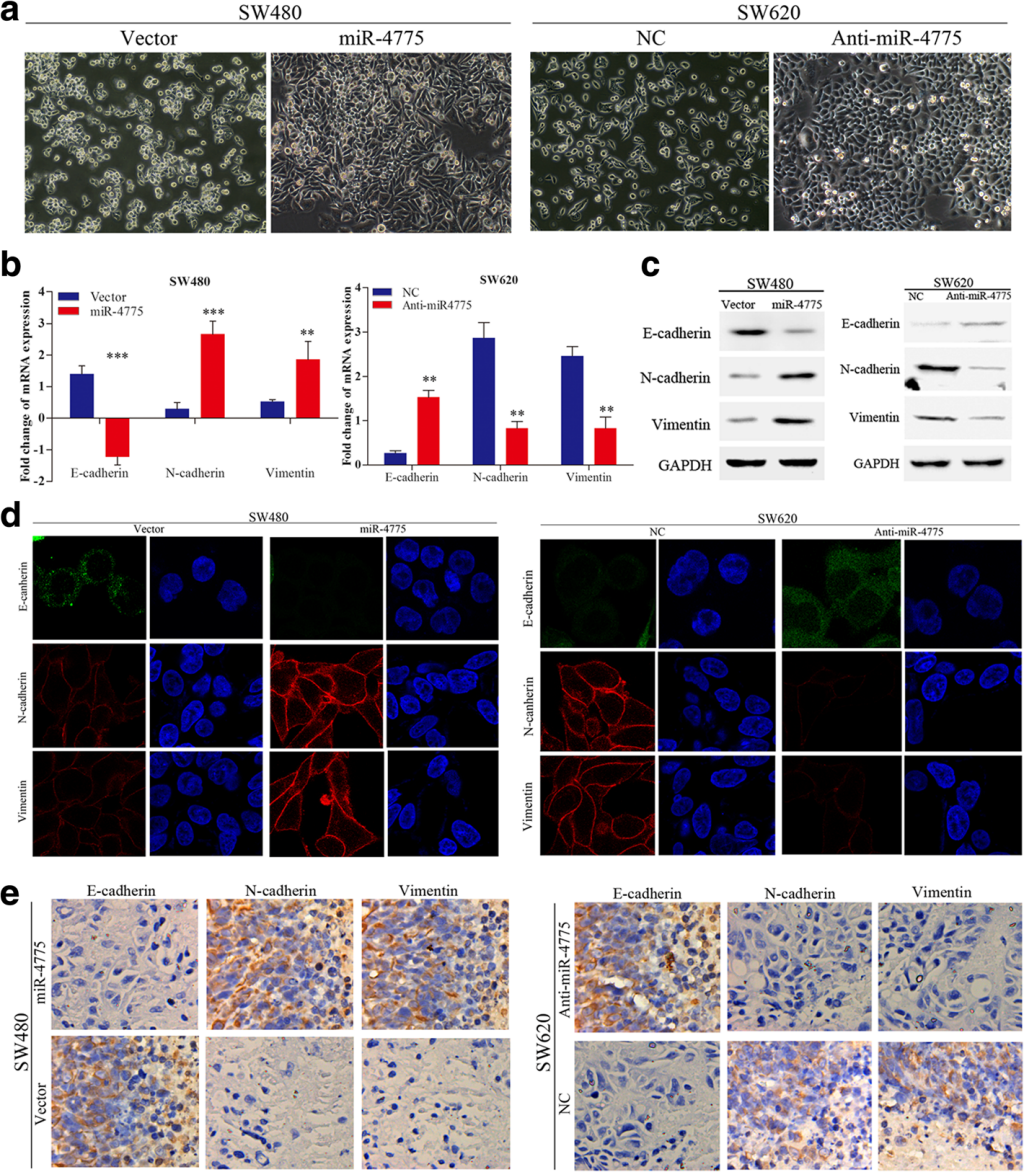
miR-4775 induces EMT in CRC. **a** The cell phenotype is significantly altered after transfection of miR-4775 mimics or anti-miR-4775 mimics into SW480 or SW620, respectively, compared with their control groups (magnification × 200). **b** Fold changes in EMT marker mRNAs upon miR-4775 overexpression or downregulation compared with their control groups (**p < 0.01, ***p < 0.001). **c** Western blotting analyses of EMT markers in cells overexpressing or knocked down for miR-4775 and control cells. **d** Immunofluorescence staining analysis of EMT markers in cells overexpressing or knocked down for miR-4775 and control cells. **e** IHC staining analysis of EMT markers in cells overexpressing or knocked down for miR-4775 and their control subcutaneous xenograft tumors in nude mice (magnification × 200)

Interestingly, we also found that overexpressing miR-4775 could induce cells to adopt a spindle-like fibroblastic cell phenotype; in contrast, downregulating miR-4775 reversed this change (Fig. 5a). Thus, we hypothesized that miR-4775 could induce CRC cells to undergo EMT. qPCR and western blot assays showed that overexpressing or knocking down miR-4775 downregulated or upregulated, respectively, E-cadherin expression while upregulating or downregulating, respectively, N-cadherin and vimentin mRNA and protein levels (Fig. 5b and c). Immunofluorescence staining also revealed that miR-4775 overexpression or knockdown increased or decreased, respectively, E-cadherin expression and decreased or increased, respectively, N-cadherin and vimentin expression (Fig. 5d). More importantly, IHC staining of EMT markers in the xenografted tumors of nude mice further confirmed that overexpressing or knocking down miR-4775 promoted or inhibited, respectively, EMT in vivo (Fig. 5e). Collectively, these results indicate that miR-4775 promotes EMT in CRC cells in vivo.

### miR-4775 is a novel activator of the Smad7/TGFβ pathway

**Fig. 6:**
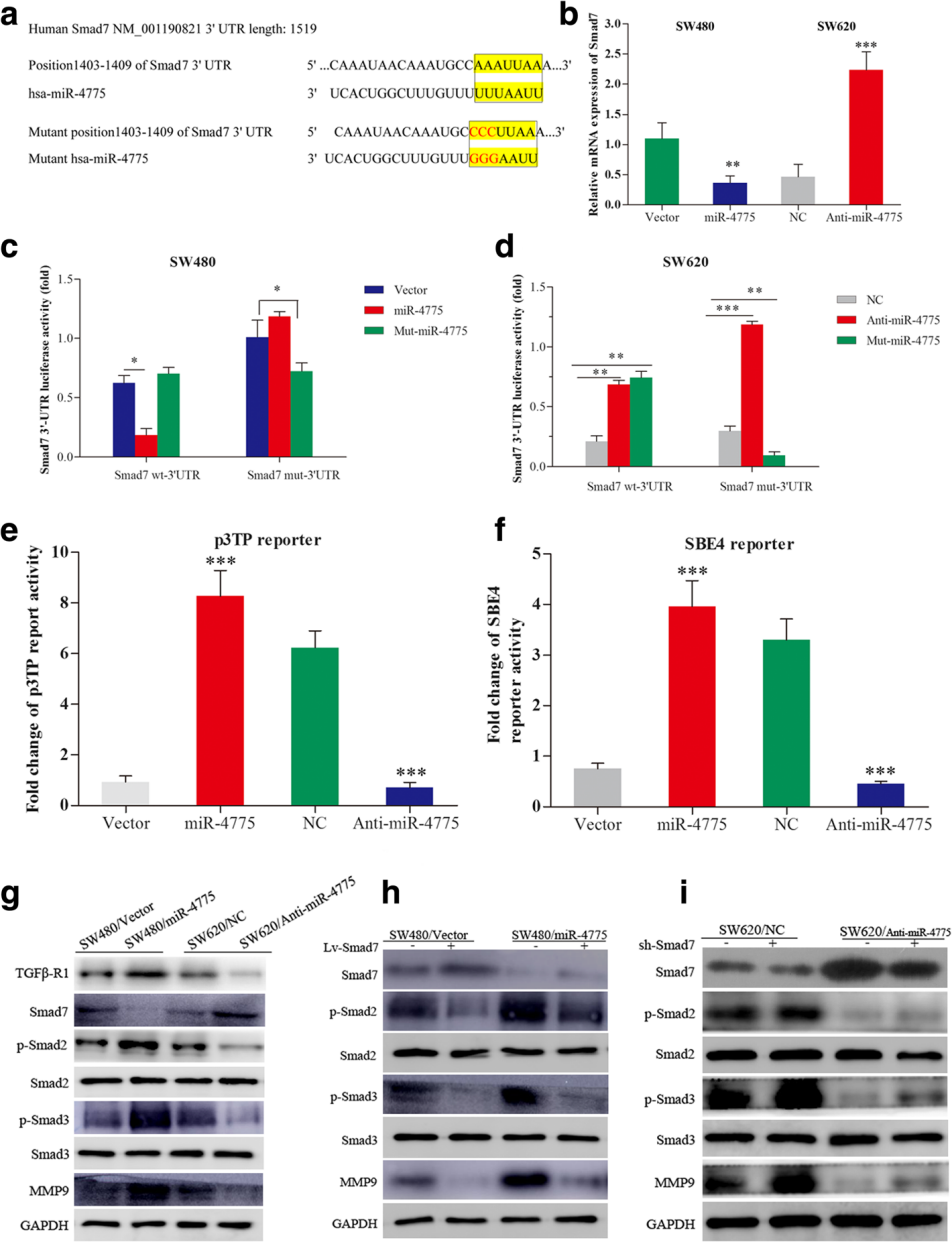
miR-4775 regulates the TGFβ pathway by directly repressing Smad7. **a** Potential miR-4775 binding sites in the Smad7 3′UTR and mutated miR-4775 binding sites in the Smad7 3′UTR. **b** Smad7 mRNA changes upon miR-4775 overexpression or downregulation (**p < 0.01;***p < 0.001). **c**–**d** Effects of miR-4775 overexpression or knock down on luciferase dual reporter activity with the wild-type and mutated Smad7 3′UTR. **e** p3TP and **f** SBE4 reporter assay to analyze the roles of miR-4775 overexpression or knock down on activation of TGFβ signaling. **g** Western blot analyses of Smad7/TGFβ pathway proteins after upregulation or downregulation of miR-4775 expression. **h** Effects of Smad7 overexpression on the protein levels of TGFβ pathway-associated markers in miR-4775-overexpressing and control cells. **i** Effects of decreases in Smad7 on the protein levels of TGFβ pathway-associated markers in cells knocked down for miR-4775 and the corresponding control group

To identify target genes of miR-4775, we performed bioinformatics analysis with the public algorithm TargetScan7.0 (http://www.targetscan.org/). We screened cell invasion-, metastasis- and EMT pathway-related genes that have 3′UTRs containing the miR-4775-binding site, and Smad7 was identified as a candidate (Fig. 6a). qPCR analyses showed that overexpressing or knocking down miR-4775 down- or upregulated Smad7 mRNA levels in SW480 or SW620 cells (Fig. 6b). Furthermore, a wild-type Smad7 3′UTR luciferase reporter vector was constructed and transfected, and miR-4775 overexpression or knockdown caused lower or higher Smad7 3′UTR luciferase activity, respectively (Fig. 6c). To demonstrate whether miR-4775 directly promotes degradation of the Smad7 3′UTR, we constructed luciferase reporter plasmids with a mutated Smad7 3′UTR and co-transfected them with miR-4775, anti-miR-4775 and their corresponding negative control vectors. The data shown in Fig. 6c indicate that overexpressing miR-4775 decreased luciferase activity in cells harboring the wild-type Smad7 3′UTR construct but not in cells carrying the mutant Smad7 3′UTR (Fig. 6c). However, mutant miR-4775 could decrease the luciferase activity of the mutant Smad7 3′UTR construct, whereas knocking down miR-4775 expression increased the luciferase activity of the wild-type Smad7 3′UTR but not that of the mutant Smad7 3′UTR (Fig. 6d). These data suggest that miR-4775 directly downregulates Smad7 expression by recognizing its 3′UTR. We further investigated whether miR-4775 regulates TGFβ signaling by evaluating the TGFβ/active-responsive SBE4-luc reporter and p3TP reporter activities. Overexpressing or knocking down expression of miR-4775 increased or decreased, respectively, SBE4-luc reporter and p3TP reporter activities compared with their control groups (Fig. 6e and f). Taken together, these results further indicate that miR-4775 activates the TGFβ pathway by directly downregulating Smad7 expression.

Subsequently, we found that overexpressing or knocking down miR-4775 increased or decreased, respectively, TGFβ R1, p-Smad2, p-Smad3 and MMP9 protein levels and decreased or increased, respectively, the Smad7 protein level (Fig. 6g). Furthermore, overexpressing Smad7 partially reversed the increase in p-Smad2, p-Smad3 and MMP9 protein levels caused by miR-4775 overexpression (Fig. 6h), whereas knocking down Smad7 partially reversed the decreases in p-Smad2, p-Smad3 and MMP9 protein levels caused by miR-4775 downregulation (Fig. 6i). Altogether, these results demonstrate that miR-4775 activates the Smad7/TGFβ pathway in CRC cells.

### miR-4775 promotes the invasion and metastasis of CRC cells by downregulating Smad7 in vitro and in vivo

**Fig. 7:**
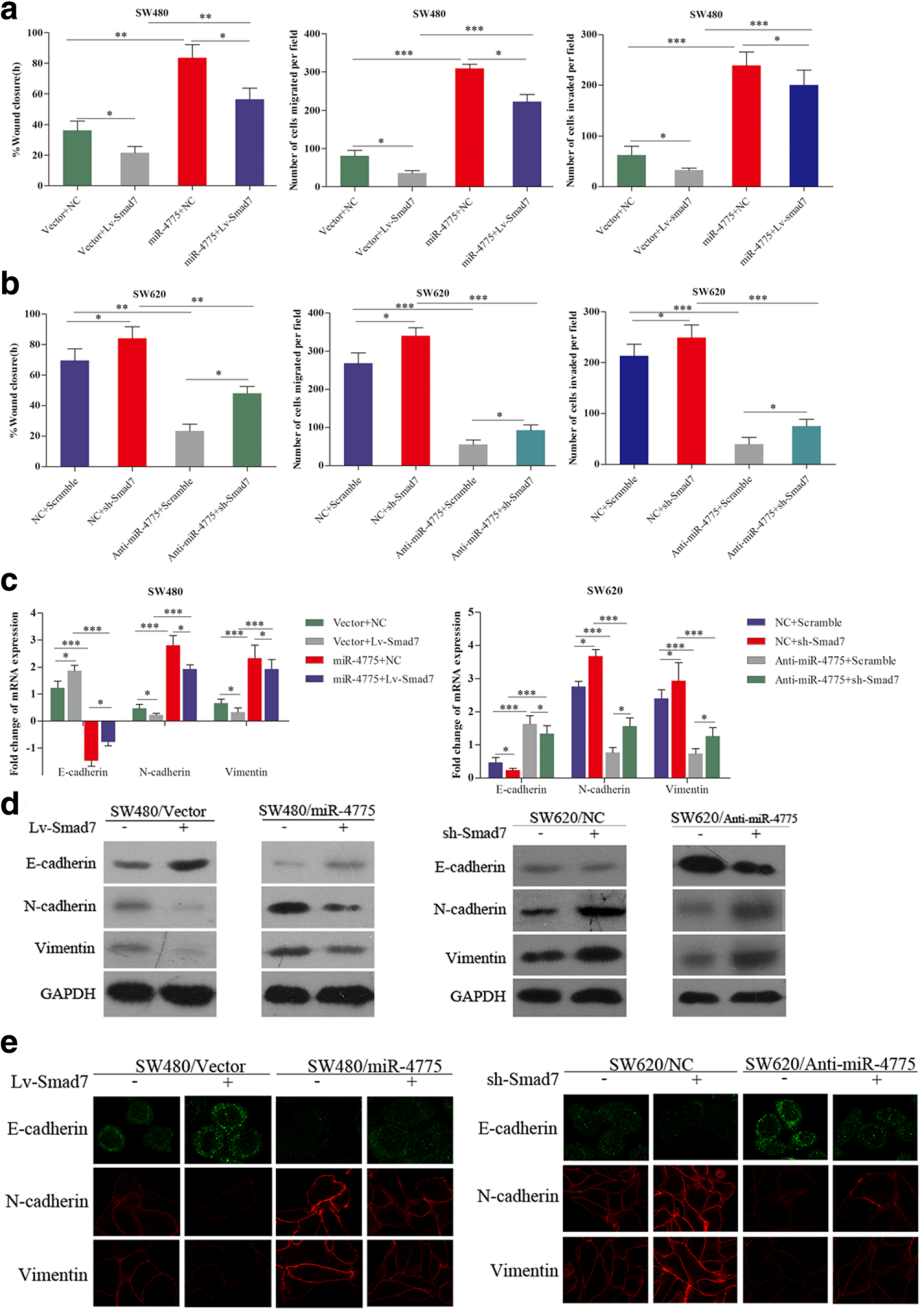
Roles of Smad7 in miR-4775-mediated tumor progression in vitro. **a** Overexpressing Smad7 partially weakened the promoting effect of miR-4775 upregulation on SW480 cell wound healing, migration and invasion abilities compared with the control groups (*p < 0.05;**p < 0.01;***p < 0.001). **b** Knocking down Smad7 partially inhibited the inhibitory effect of miR-4775 downregulation on SW620 cell wound healing, migration and invasion abilities compared with the control group (*p < 0.05;**p < 0.01;***p < 0.001). **c** Overexpressing or knocking down Smad7 partially reversed the effects of miR-4775 overexpression or knock down on the mRNA levels of EMT markers. **d** Overexpressing or knocking down Smad7 partially reversed the effects of miR-4775 overexpression or knock down on the protein levels of EMT markers. **e** Immunofluorescence staining analysis of EMT markers with regard to Smad7 overexpression or knockdown in cells with overexpressing or knocked down for miR-4775 and the corresponding control cells

Given the above effects of miR-4775 on the Smad7/TGFβ pathway, we further investigated whether miR-4775 could promote CRC invasion and metastasis by directly degrading Smad7. We found that the effects of overexpressing or knocking down miR-4775 on SW480 or SW620 cell wound healing, migration and invasion were partially weakened or strengthened by overexpressing or repressing Smad7 (Fig. 7a and b). With regard to both mRNA and protein levels of EMT markers, overexpressing or knocking down Smad7 partially weakened or restored, respectively, the downregulation or upregulation of E-cadherin expression and the upregulation or downregulation of N-cadherin and vimentin expression caused by overexpressing or knocking down miR-4775 (Fig. 7c–e). These results suggest that miR-4775 promotes CRC cell migration, invasion and EMT by degrading the Smad7 3′UTR in vitro.

**Fig. 8:**
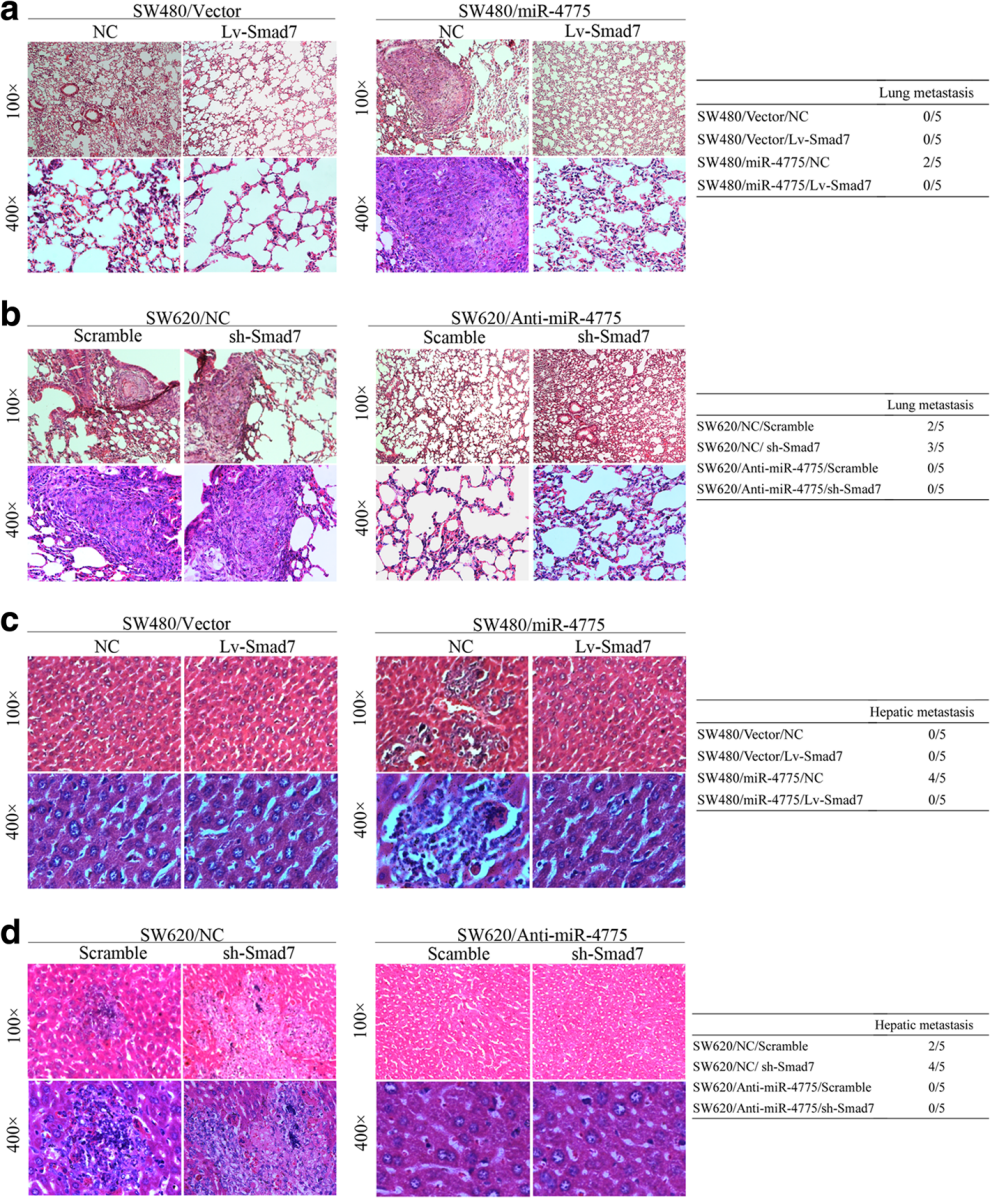
Effects of Smad7 on miR-4775-mediated lung and liver metastases in nude mice. **a**–**b** Overexpressing or knocking down Smad7 inhibited or promoted lung metastases caused by miR-4775 upregulation or downregulation in nude mice (***p < 0.001). **c**–**d** Overexpressing or knocking down Smad7 decreased or increased the liver metastasis triggered by miR-4775 overexpression or knockdown in vivo (***p < 0.001)

Next, we injected these cells into the tail veins of nude mice to evaluate whether miR-4775 enhances the metastatic ability via Smad7 repression in vivo. We found that overexpressing or knocking down Smad7 also partially weakened or restored, respectively, the lung and liver metastasis capacity resulting from overexpressing or knocking down miR-4775 (Fig. 8a–d). Accordingly, our in vitro and in vivo assays demonstrate that miR-4775 increases the invasion and metastasis abilities of CRC cells by downregulating Smad7.

## Discussion

We found that miR-4775 was upregulated in CRC tissues, predicting poor prognosis for CRC patients. Indeed, miR-4775 overexpression promoted CRC cell invasion, metastasis and EMT in vitro and in vivo, highlighting the important role of miR-4775 in tumorigenesis and the development of CRC. Furthermore, the present study verified that miR-4775 regulates Smad7/TGFβ pathway activation. The high risk of tumor recurrence and metastasis suggests that CRC patients with high miR-4775 expression and Smad7/TGFβ pathway activation should receive individualized and more aggressive treatment after tumor resection.

Although we have achieved a better understanding of the genetic alterations in CRC in recent years, the prognosis of CRC patients remains poor because of cancer cell invasion and distant metastasis [24], which result from the inherent heterogeneity and complex gene interactions of CRC [25]. Accordingly, discovering new biological mechanisms and novel regulators of CRC will provide a better approach for controlling CRC development [4]. In the present, we identified miR-4775 as a driver of CRC progression. These findings are consistent with a previous report that miR-4775 acts as an onco-miRNA and promotes breast cancer development via a dual effect on the ERBB2/Her2 gene [8]. It is widely accepted that tumor invasion and metastasis are the main driving forces of relapse [2], and herein, high miR-4775 expression was positively correlated with TNM stage. Furthermore, the level of miR-4775 expression was clearly higher in the tumor recurrence group than in the non-recurrence group. Most importantly, high expression of miR-4775 predicted poor OS in the tumor recurrence group, though no such significant correlation was found in the non-recurrent group. Overexpressing miR-4775 increased CRC cell wound healing, migration and invasion ability and enhanced CRC cell lung and liver metastasis in nude mice. EMT has been widely reported as a critical step in tumor invasion and metastasis [26]. For example, LncRNA-ATB was shown to induce hepatocellular carcinoma EMT and invasion by competitively binding to and inhibiting miR-200 family members and then upregulating ZEB1 and ZEB2 [27]. We also verified that overexpressing miR-4775 induces EMT in CRC cells, with changes in cell morphology, upregulation of the mesenchyme markers N-cadherin and vimentin, and downregulation of the epithelial cell marker E-cadherin. These findings further support our previous hypothesis that miR-4775 promotes the progression and recurrence of CRC in patients by increasing the invasive and metastatic abilities of CRC cells.

TGFβ signaling is widely reported to be involved in several biological processes, such as tumor cell growth, apoptosis, adhesion, migration, and differentiation [28]. More importantly, in advanced stages of several epithelial cancers, TGFβ functions as a potential activator of cancer progression and metastasis by promoting EMT [29]. TGFβ signaling is activated by the interaction of heterodimeric transmembrane serine/threonine kinase complexes, including type I (TGFβ RI) and type II (TGFβ RII) receptors, with cytokines [30]. In the inactivation process, Smad7 blocks R-Smad phosphorylation by occupying the catalytic domain of TGFβ RI. Inhibiting Smad7 leads to activation of R-Smads via phosphorylation through TGFβ RI. Activated R-Smads (p-Smad2 and p-Samd3) form a complex with Smad4, which translocates to the nucleus and regulates the transcription of specific target genes [31]. In the present study, the promoting function of high miR-4775 expression was weakened by overexpression of Smad7, and high miR-4775 expression was generally found to be concomitant with low Smad7 and high p-Smad2 and p-Smad3. Smad7 levels decreased and p-Smad2 and p-Smad3 levels increased gradually with enhanced miR-4775 expression. These findings indicate that the underlying mechanism by which miR-4775 promotes CRC progression is related to canonical activation of the Smad7/TGFβ pathway. In addition, other miRNAs have been described as affecting EMT in CRC by targeting the Smad7/TGFβ pathway, including miR1269, miR224 and miR200c [7, 19, 26]. Nonetheless, it needs to be further explored whether miR-4775 has synergistic effects with these reported miRNAs on EMT in CRC and whether the impact of miR-4775 is more important than that of these miRNAs.

## Conclusions

In summary, our present study demonstrates the critical roles of miR-4775 and the Smad7/TGFβ pathway in the progression of CRC. Moreover, we identified a novel activation mechanism of the TGFβ pathway in CRC, providing a new treatment strategy for improving the prognosis of these patients.

## Additional files

Additional file 1: Table S1. Clinic-pathologic characteristics of 544 colorectal cancer patients. (DOCX 20 kb)
Additional file 2: Table S2. Data of antibodies used in the present research. (DOCX 17 kb)
Additional file 3: Table S3. miR-4775 expression with relation to metastasis in 544 CRC patients. (DOCX 18 kb)
Additional file 4: Table S4. Multivariate Cox proportional hazard analyses of miR-4775 expression, T stage, Lymph node metastasis and distant metastasis in association with DFS and OS in 544 CRC patients. (DOCX 16 kb)
Additional file 5: Table S5. Correlations between miR-4775 expression and E-cadherin, N-cadherin and vimentin staining in tumor tissues from 544 CRC patients. (DOCX 16 kb)
Additional file 6: Table S6. Correlations between miR-4775 expression and Smad7, p-Smad2, p-Smad3 staining in tumor tissues from 544 CRC patients. (DOCX 16 kb)

## Abbreviations

3′-UTR: 3′-untranslated regions
CRC: Colorectal cancer
EMT: Epithelial to mesenchymal transition
GAPDH: Glyceraldehyde-3-phosphate dehydrogenase
IHC: Immunohistochemistry
ISH: In situ hybridization
TGFβ: Transforming growth factor-β
