# Supplementary material for: miR-4775 promotes colorectal cancer invasion and metastasis via the Smad7/TGFβ-mediated epithelial to mesenchymal transition
Source: Mol Cancer. 2017 Jan 17;16:12. doi: 10.1186/s12943-017-0585-z (PMC5240405; doi:10.1186/s12943-017-0585-z)
Supplement: Additional file 1: Table S1. — Clinic-pathologic characteristics of 544 colorectal cancer patients. (DOCX 20 kb) [file 12943_2017_585_MOESM1_ESM.docx]

**Table S1.** Clinic-pathologic characteristics of 544 colorectal cancer patients.

| **Characteristics** | **No. of patients (%)** | **Relapse** | |
| --- | --- | --- | --- |
|  |  | **No n=327 (%)** | **Yes n=217 (%)** |
| Age |  |  |  |
| <65 | 208 (38.2) | 130 (39.8) | 78 (35.9) |
| ≥65 | 336 (61.8) | 197 (60.2) | 139 (64.1) |
| Gender |  |  |  |
| Female | 264 (48.5) | 165 (49.5) | 99 (45.6) |
| Male | 280 (51.5) | 162 (50.5) | 118 (54.4) |
| Location |  |  |  |
| Right | 124 (22.8) | 76 (23.2) | 48 (22.1) |
| Transverse | 33 (6.1) | 20 (6.1) | 13 (6.0) |
| Left | 387 (71.1) | 231 (70.7) | 156 (71.9) |
| pT stage |  |  |  |
| pT1 | 23(4.2) | 20(6.1) | 3(1.4) |
| pT2 | 94 (17.3) | 72 (22.0) | 22 (10.1) |
| pT3 | 236 (43.4) | 152(46.5) | 84 (38.7) |
| pT4 | 191 (35.1) | 83 (25.4) | 108 (49.8) |
| pN stage |  |  |  |
| N0 | 314(57.7) | 216(66.1) | 98(45.1) |
| N1 | 164(30.1) | 87(26.6) | 77(35.5) |
| N2 | 66(12.1) | 24(7.3) | 42(19.4) |
| pM stage |  |  |  |
| M0 | 504 | 317(96.9) | 187(86.2) |
| M1 | 40 | 10(3.1) | 30(13.8) |
| AJCC stage |  |  |  |
| I | 93(17.1) | 76 (23.2) | 17 (7.8) |
| II | 208 (38.2) | 136(41.6) | 72 (33.2) |
| III | 204 (37.5) | 106 (32.4) | 98 (45.2) |
| IV | 39(7.2) | 9(2.8) | 30(13.8) |
| Differentiation |  |  |  |
| Well | 116 (28.0) | 71 (21.7) | 45 (20.7) |
| Moderate | 336 (41.9) | 201 (61.5) | 135 (62.2) |
| Poor | 92 (30.1) | 55 (16.8) | 37 (17.1) |
| miR-4775 |  |  |  |
| Low expression | 142(26.1) | 129(39.4) | 13(6.0) |
| High expression | 402(73.9) | 198(60.6) | 204(94.0) |

AJCC ,American Joint Committee on Cancer.
