# Supplementary material for: miR-4775 promotes colorectal cancer invasion and metastasis via the Smad7/TGFβ-mediated epithelial to mesenchymal transition
Source: Mol Cancer. 2017 Jan 17;16:12. doi: 10.1186/s12943-017-0585-z (PMC5240405; doi:10.1186/s12943-017-0585-z)
Supplement: Additional file 2: Table S2. — Data of antibodies used in the present research. (DOCX 17 kb) [file 12943_2017_585_MOESM2_ESM.docx]

**Table S2.** Data of antibodies used in the present research.

| Antibody | WB | IHC | IF | Specificity | Company |
| --- | --- | --- | --- | --- | --- |
| TGFβRI(#3712) | 1:1000 | - | - | Rabbit  monoclonal | Cell Signaling Technology |
| MMP9(#13667) | 1:1000 | - | - | Rabbit monoclonal | Cell Signaling Technology |
| E-cadherin (#-14479) | 1:1000 | 1:50 | 1:50 | Mouse monoclonal | Cell Signaling Technology |
| N-cadherin (#13116) | 1:1000 | 1:125 | 1:200 | Rabbit polyclonal | Cell Signaling Technology |
| Vimentin (#-5741) | 1:100 | 1:100 | 1:100 | Rabbit monoclonal | Cell Signaling Technology |
| Smad7 (sc-11392) | 1:100 | 1:200 | 1:500 | Rabbit  polyclonal | Santa Cruz  Biotechnology |
| p-Smad2 (ab188334) | 1:10000 | 1:500 | - | Rabbit Monoclonal | Abcam |
| Smad2 (ab40855) | 1:10000 | - | - | Rabbit Monoclonal | Cell Signaling Technology |
| p-Smad3 (ab52903) | 1:2000 | 1:500 | - | Rabbit Monoclonal | Abcam |
| Smad3 (ab40854) | 1:10000 | - | - | Rabbit monoclonal | Abcam |
| GAPDH (ab181602) | 1:10000 | - | - | Rabbit monoclonal | Abcam |
