# Supplementary material for: miR-4775 promotes colorectal cancer invasion and metastasis via the Smad7/TGFβ-mediated epithelial to mesenchymal transition
Source: Mol Cancer. 2017 Jan 17;16:12. doi: 10.1186/s12943-017-0585-z (PMC5240405; doi:10.1186/s12943-017-0585-z)
Supplement: Additional file 3: Table S3. — miR-4775 expression with relation to metastasis in 544 CRC patients. (DOCX 18 kb) [file 12943_2017_585_MOESM3_ESM.docx]

Table S3. miR-4775 expression with relation to metastasis in 544 CRC patients.

| **Characteristics** | **No. of patients (%)** | **miR-4775g expression** | | ***P*** * |
| --- | --- | --- | --- | --- |
|  |  | **Low n= 142 (%)** | **High n=402 (%)** |  |
| Age  <65  ≥65  Gender  Female  Male  Location  Ascend  Transverse  Descend  Sigmoid  Rectal  pT stage  T1  T2  T3  T4  Lymph node metastasis | 208(38.2)  336(61.8)  264(48.5)  280(51.5)  124(22.8)  33(6.1)  29(5.3)  145(26.7)  213(39.2)  23(4.2)  94(17.3)  236(43.4)  191(35.1) | 52(36.6)  90(63.4)  77(54.2)  65(45.8)  39(27.5)  10(7.0)  3(2.1)  38(26.8)  52(36.6)  8(5.6)  33(23.2)  67(47.2)  34(23.9) | 156(38.8)  246(61.2)  203(50.5)  199(49.5)  85(21.1)  23(5.7)  26(6.5)  107(26.6)  161(40.0)  15(3.7)  61(15.2)  169(42.0)  157(39.1) | 0.688  0.494  0.185  0.006 |
| N0 | 314(57.7) | 99 (69.7) | 215 (53.5) | <0.001 |
| N1 | 164(30.1) | 38(26.8) | 126(31.3) |  |
| N2 | 66(12.1) | 5(3.5) | 61(15.2) |  |
| Distant metastasis |  |  |  |  |
| M0 | 504(92.6) | 137(96.5) | 367(91.3) | 0.042 |
| M1 | 40(7.4) | 5 (3.5) | 35(8.7) |  |
