# Supplementary material for: miR-4775 promotes colorectal cancer invasion and metastasis via the Smad7/TGFβ-mediated epithelial to mesenchymal transition
Source: Mol Cancer. 2017 Jan 17;16:12. doi: 10.1186/s12943-017-0585-z (PMC5240405; doi:10.1186/s12943-017-0585-z)
Supplement: Additional file 4: Table S4. — Multivariate Cox proportional hazard analyses of miR-4775 expression, T stage, Lymph node metastasis and distant metastasis in association with DFS and OS in 544 CRC patients. (DOCX 16 kb) [file 12943_2017_585_MOESM4_ESM.docx]

Table S4. Multivariate Cox proportional hazard analyses of miR-4775 expression, T stage, Lymph node metastasis and distant metastasis in association with DFS and OS in 544 CRC patients.

| **Factors** | **Multivariate Analyses(DFS)** | |  | **Multivariate Analyses(OS)** | |
| --- | --- | --- | --- | --- | --- |
|  | **HR (95% CI)** | ***P**** |  | **HR (95% CI)** | ***P**** |
| T(T3-4 vs T1-2) | 1.56(1.34-2.24) | <0.001 |  | 2.34(1.68-4.35) | <0.001 |
| Lymph node metastasis | 1.41 (1.17-1.70) | <0.001 |  | 1.34(1.11-1.61) | 0.002 |
| Distant metastasis | 2.71 (1.80-4.06) | <0.001 |  | 2.71(1.80-4.06) | <0.001 |
| miR-4775 expression | 10.19(5.01-20.75) | <0.001 |  | 10.52(5.16-21.42) | <0.001 |

**P* < 0.05 indicates significance; HR, hazard ratio; CI, confidence interval.
