# Supplementary material for: miR-4775 promotes colorectal cancer invasion and metastasis via the Smad7/TGFβ-mediated epithelial to mesenchymal transition
Source: Mol Cancer. 2017 Jan 17;16:12. doi: 10.1186/s12943-017-0585-z (PMC5240405; doi:10.1186/s12943-017-0585-z)
Supplement: Additional file 5: Table S5. — Correlations between miR-4775 expression and E-cadherin, N-cadherin and vimentin staining in tumor tissues from 544 CRC patients. (DOCX 16 kb) [file 12943_2017_585_MOESM5_ESM.docx]

**Table S5** Correlations between miR-4775 expression and E-cadherin ,N-cadherin and Vimentin staining in tumor tissues from 544 CRC patients.

| IHC staining | n | miR-4775 expression | | | p* |
| --- | --- | --- | --- | --- | --- |
|  |  | Low (n=142) | High(n=402) | |  |
| E-cadherin |  |  | |  |  |
| Negative/Weak | 414 | 49 | | 365 | <0.001 |
| Strong | 130 | 93 | | 37 |  |
| N-cadherin |  |  | |  |  |
| Negative/Weak | 137 | 95 | | 42 | <0.001 |
| Strong | 407 | 47 | | 360 |  |
| Vimentin |  |  | |  |  |
| Negative/Weak | 177 | 76 | | 101 | <0.001 |
| Strong | 467 | 66 | | 301 |  |

**p*<0.05 indicates a significant relationship among the variables
