# Supplementary material for: miR-4775 promotes colorectal cancer invasion and metastasis via the Smad7/TGFβ-mediated epithelial to mesenchymal transition
Source: Mol Cancer. 2017 Jan 17;16:12. doi: 10.1186/s12943-017-0585-z (PMC5240405; doi:10.1186/s12943-017-0585-z)
Supplement: Additional file 6: Table S6. — Correlations between miR-4775 expression and Smad7, p-Smad2, p-Smad3 staining in tumor tissues from 544 CRC patients. (DOCX 16 kb) [file 12943_2017_585_MOESM6_ESM.docx]

**Table S6** Correlations between miR-4775 expression and Smad7, p-Smad2, p-Smad3 staining in tumor tissues from 544 CRC patients.

| IHC staining | n | miR-4775 expression | | | p* |
| --- | --- | --- | --- | --- | --- |
|  |  | Low (n=142) | High(n=402) | |  |
| Smad7 |  |  | |  |  |
| Negative/Weak | 374 | 19 | | 355 | <0.001 |
| Strong | 170 | 123 | | 47 |  |
| p-Smad2 |  |  | |  |  |
| Negative/Weak | 247 | 125 | | 122 | <0.001 |
| Strong | 297 | 17 | | 280 |  |
| p-Smad3 |  |  | |  |  |
| Negative/Weak | 237 | 106 | | 131 | <0.001 |
| Strong | 307 | 36 | | 271 |  |

**p*<0.05 indicates a significant relationship among the variables
